# A two arm randomized controlled trial comparing the short and long term effects of an elimination diet and a healthy diet in children with ADHD (TRACE study). Rationale, study design and methods

**DOI:** 10.1186/s12888-020-02576-2

**Published:** 2020-05-27

**Authors:** Annick Bosch, Margreet Bierens, Ardine G. de Wit, Verena Ly, Jessica van der Velde, Heleen de Boer, Gerry van Beek, Danielle Appelman, Sacha Visser, Lisa Bos, Jolanda van der Meer, Niki Kamphuis, Jos M. T. Draaisma, Rogier Donders, Gigi H. H. van de Loo-Neus, Pieter J. Hoekstra, Marco Bottelier, Alejandro Arias-Vasquez, Helen Klip, Jan K. Buitelaar, Saskia W. van den Berg, Nanda N. Rommelse

**Affiliations:** 1 Karakter, Child and Adolescent Psychiatry, Reinier Postlaan 12, 6525 GC Nijmegen, the Netherlands; 2 National Institute for Public Health and the Environment (RIVM), Bilthoven, the Netherlands; 3 Julius Center for Health Sciences and Primary Care, University Medical Center Utrecht, Utrecht, the Netherlands; 4 Leiden University, Institute of Psychology and Leiden Institute for Brain and Cognition, Leiden, The Netherlands; 5 Accare, Child and Adolescent Psychiatry, Groningen, the Netherlands; 6 Triversum - GGZ-NHN, Child and Adolescent Psychiatry, Alkmaar, the Netherlands; 7 Freelance dietician, Velp, the Netherlands; 8 De Bascule, Center for Child and Adolescent Psychiatry, Amsterdam, The Netherlands; 9 Department of Pediatrics, Radboud University Medical Center Amalia Children’s hospital, Nijmegen, the Netherlands; 10 Department for Health Evidence, Radboud University Medical Center, Nijmegen, the Netherlands; 11 Department of Child and Adolescent Psychiatry, University of Groningen, University Medical Center Groningen, Groningen, the Netherlands; 12 Department of Psychiatry, Radboud University Medical Center, Nijmegen, The Netherlands; 13 Department of Human Genetics, Donders Institute for Brain, Cognition and Behavior, Radboud University Medical Center, Nijmegen, The Netherlands; 14 Department of Cognitive Neuroscience, Donders Institute for Brain, Cognition and Behavior, Radboud University Medical Center, Nijmegen, The Netherlands

**Keywords:** ADHD, Children, Dietary treatment, Short and long-term effects, Cost-effectiveness

## Abstract

**Background:**

Food may trigger Attention-Deficit/Hyperactivity Disorder (ADHD) symptoms. Therefore, an elimination diet (ED) might be an effective treatment for children with ADHD. However, earlier studies were criticized for the nature of the control group, potential confounders explaining the observed effects, unsatisfactory blinding, potential risks of nutritional deficiencies and unknown long term and cost-effectiveness. To address these issues, this paper describes the rationale, study design and methods of an ongoing two arm randomized controlled trial (RCT) comparing the short (5 week) and long term (1 year) effects of an elimination diet and a healthy diet compared with care as usual (CAU) in children with ADHD.

**Methods:**

A total of *N* = 162 children (5–12 years) with ADHD will be randomized to either an ED or a healthy diet. A comparator arm including *N* = 60 children being solely treated with CAU (e.g. medication) is used to compare the effects found in both dietary groups. The two armed RCT is performed in two youth psychiatry centers in the Netherlands, with randomization within each participating center. The primary outcome measure is response to treatment defined as a ≥ 30% reduction on an ADHD DSM-5 rating scale (SWAN) and/or on an emotion dysregulation rating scale (SDQ: dysregulation profile). This is assessed after 5 weeks of dietary treatment, after which participants continue the diet or not. Secondary outcome measures include the Disruptive Behavior Diagnostic Observational Schedule (DB-DOS), parent and teacher ratings of comorbid symptoms, cognitive assessment (e.g. executive functions), school functioning, physical measurements (e.g. weight), motor activity, sleep pattern, food consumption, nutritional quality of the diet, adherence, parental wellbeing, use of health care resources and cost-effectiveness. Assessments take place at the start of the study (T0), after five weeks (T1), four months (T2), eight months (T3) and 12 months of treatment (T4). T0, T1 and T4 assessments take place at one of the psychiatric centers. T2 and T3 assessments consist of filling out online questionnaires by the parents only.

**Discussion:**

This RCT will likely contribute significantly to clinical practice for ADHD by offering insight into the feasibility, nutritional quality, (cost-)effectiveness and long term effects of dietary treatments for ADHD.

**Trial registration:**

www.trialregister.nl, NTR5434. Registered at October 11th, 2015.

## Background

Attention-Deficit/Hyperactivity Disorder (ADHD) is a neuropsychiatric disorder that affects about 7.2% of children and adolescents [1]. Both genetic and environmental factors contribute to the development of ADHD [2]. Treatment for ADHD includes psychoeducation, child behavioral interventions, parent training and medication. However, long-term beneficial effects of these treatments have not been convincingly established [3, 4], and there is also a growing concern about increasing prescription rates of medication and potential long-term side-effects [5–7]. Therefore, the present study focuses on a non-pharmacological intervention for children with ADHD, namely a dietary intervention.

There is a growing awareness that diet may play a role in psychological well-being and psychiatric disorders like ADHD [8–10]. Results of two meta-analyses and one recent review study [11–13] demonstrate that the so-called elimination diet (ED) showed the largest effects. For 30% of children an ED was effective in reducing ADHD symptoms [14]. The rationale for an ED is that a patient with ADHD may show adverse reactions to certain types of food. The ED involves a temporary (two-five weeks) total change of diet in which the patient is allowed to eat only a limited number of different hypo-allergenic foods (e.g. rice, turkey, lettuce and pears). After a significant reduction of ADHD symptoms, a 12 month reintroduction phase is needed to find out specifically which products trigger ADHD symptoms.

However, the ED studies showed large disparity in effect sizes. Results of studies which reported larger effect sizes [14] were questioned for several reasons [15–17]. A main concern was the nature of the control condition. This condition involved non-obligatory healthy food advice with no constraints and instructions for changing dietary habits compared with the ED group. Therefore, the control group differed essentially from the ED group regarding time investment, impact on family structure and parental treatment expectations. In addition, given that observational studies showed that unhealthy dietary patterns are associated with a higher risk of ADHD [18, 19], it remained unclear what caused the observed intervention-effect: specific elimination of certain foods or an overall general improvement of nutritional quality of the child’s diet. Thus far, no studies have examined the effects of an ED and a healthy diet (i.e. with different impact on nutritional intake), but with comparable impact on non-specific factors (e.g. time investment). By creating equivalent non-specific factors in both groups, it would be possible to examine the impact of nutritional intake only. A second concern was that the long-term effects (e.g. after one year) and feasibility of an ED are unknown. There are concerns about, for example, the nutritional quality of the diet, potential risks of nutritional deficiencies in the long term or the high burden it can place on the family. A third concern was that there are no studies reporting on the cost-effectiveness of an ED. A dietary treatment may be cost-effective: treatment expenses may be limited to once-only costs for a relatively short trajectory (in contrast with for example medication). A final concern was that earlier studies suffered from unsatisfactory blinding. Primary outcome measurements were based on a blinded pediatrician’s clinical judgment who based his judgment on the non-blinded information provided by parents. Expectation bias originating from parents and/or pediatricians may have influenced the outcomes.

To address these issues, the ‘Treatment of ADHD with Care as usual versus an Elimination diet’ (TRACE) study has been developed. This is the first RCT (two armed, 50:50 ratio) to determine the short- and long-term effectiveness and cost-effectiveness of an ED compared to an active control group (a healthy diet). Care As Usual (CAU) is included as a comparator arm. During the initial 5 weeks of the study, participants receiving a dietary treatment are not allowed to start any other treatment. Also, medication prior to the dietary treatment has to be discontinued no later than 1 week before start of the diet. The study will examine effects on both ADHD and emotion dysregulation, given that an ED showed equal effects on ADHD and its common comorbidity Oppositional Defiant Disorder (ODD) (i.e. children with ODD are more likely to experience emotion dysregulation problems) [14]. We will substantially improve upon previous studies by comparing an ED with a healthy diet (based on the World Health Organization (WHO) guidelines), comparing the two dietary treatments with CAU, examining the short- and long-term effects (i.e. after 5 weeks and 1 year) and cost-effectiveness, measuring the nutritional quality of the consumed diets and including blinded and objective measurements.

The primary objective of the TRACE study is to quantify the short-term (after 5 weeks) effectiveness in reducing ADHD and emotion dysregulation symptoms of an ED compared to an active control group (a healthy diet) in children aged 5–12 years with ADHD: we assume that the ED is more effective than the healthy diet. Secondary, the study aims at examining the long-term (after 1 year) effects in reducing ADHD and emotion dysregulation symptoms. Moreover, the study aims at examining the short- and long-term effects of an ED compared to an active control group (a healthy diet) in reducing secondary outcome measures related to ADHD such as cognitive outcomes, motor activity and sleep pattern. In addition, the study aims to identify potential moderators and mediators (e.g. socioeconomic status (SES), ethnicity, comorbidity) of the short- and long-term response to dietary treatments, and to examine the nutritional quality of the attained diet and health of children treated with a dietary treatment compared to CAU. Finally, the study aims to examine the cost-effectiveness of the dietary treatments compared with CAU.

## Methods

### Participants

Families are recruited at two clinical centers in the Netherlands: Karakter Child and Adolescent Psychiatry (Nijmegen) and Triversum - GGZ-NHN Child and Adolescent Psychiatry (Alkmaar). Researchers will screen files of patients (after consent of parents and/or psychiatrists) within these centers in order to check for eligibility or psychiatrists will refer the patients to the study. In addition, participants are recruited at the national level via advertisements, newspapers, the TRACE website, flyers and social media. Moreover, general practitioners, dieticians and other psychiatric institutions act as ambassadors and collaborate in the recruitment process by referring patients to the study. At the Nijmegen site, there is a team of certified clinicians who can diagnose (if necessary) participants recruited outside the participating centers.

For eligibility, participants must meet the following criteria: ADHD diagnosis according to the DSM-5 (any presentation) and 5–12 years old at the inclusion date. Comorbidities are allowed except for eating disorders (i.e. anorexia or bulimia nervosa) and diabetes mellitus. Exclusion criteria are insufficient mastery of Dutch language in parents or children; current treatment for ADHD that cannot be discontinued; significant parent-child relationship problems requiring family therapy; unwillingness to have meat or animal food products in the diet (without these products it is impossible to achieve nutritional adequacy of the overall diet for participants in the ED group). The CAU participants need to fulfill the same inclusion- and exclusion criteria.

One of the inclusion criteria includes an ADHD diagnosis. To confirm this diagnosis, children are screened by the researchers via a structured psychiatric interview with the parents based on the DSM-IV (Kiddie Schedule for Affective Disorders and Schizophrenia: K-SADS) [20]. The ADHD section and the ODD section of this interview are used. The latter is used to check for comorbidities. Also, teachers fill out the Strengths and Weaknesses of ADHD-symptoms and Normal-behaviors (SWAN) rating scale [21]. To confirm the clinical ADHD diagnosis, there should be a minimum of six out of nine symptoms present in at least one domain (i.e. hyperactivity/impulsivity or inattentiveness), based on parent and teacher ratings. Parents and teachers have to separately rate at least four out of nine symptoms in one domain. Children are not able to participate if this criterion is not met.

### Study design

#### Randomization

Originally, the TRACE study was a three arm randomized controlled trial: participants were randomized to an elimination diet, healthy diet or CAU. However, randomization across a dietary intervention versus CAU proved not feasible. Parents usually had a strong preference towards either a dietary intervention or CAU. This resulted in an extremely slow inclusion rate, a high number of drop-outs and thereby unrepresentative groups of CAU. Therefore, after inclusion of 17 participants, the design was changed into a two arm randomized controlled trial (elimination diet vs. healthy diet) with a non-randomized comparator arm (CAU). Participants interested in a dietary intervention are randomized (50:50) to either an ED or a healthy diet with randomization within each participating center. Randomization by means of minimization is performed, including sex and age as factors, resulting in four groups. Within each group, blocked randomization is used (block size eight). The non-randomized comparator arm includes children who are being treated with CAU. Researchers disclose the group allocation to parents via a sealed envelope. Participants, researchers and dieticians are aware of treatment allocation. Researchers who will code the behavior observation (see secondary outcomes) are blinded to group allocation and time of assignment.

#### 1st phase: five weeks

During the initial 5 weeks of the study, participants receiving a dietary treatment are not allowed to start treatment with medication or to receive specific psychosocial interventions other than psychoeducation in a group setting. If participants choose otherwise, this is coded as non-compliance. Similarly, participants receiving CAU are not allowed to follow a strict dietary advice. If they choose otherwise, this is coded as non-compliance. Non-compliant participants will still be assessed during the follow-up measurements.

#### 2nd phase: five weeks until 12 months after baseline

After 5 weeks, response to treatment is evaluated comparing measures of ADHD symptoms (i.e. SWAN questionnaire) and emotion dysregulation symptoms (i.e. SDQ questionnaire: dysregulation profile) before and after the 5 weeks (see Method section, primary outcome paragraph ‘respondership’). Full and partial responders (see primary outcome ‘respondership’) to the dietary treatment are invited to continue the diet in the second phase of the study until the end of the trial (12 months after baseline). Mixed responders (see primary outcome ‘respondership’) are offered to continue the diet, but are not explicitly advised as with the full and partial responders. To optimize the generalizability and feasibility of the study results to clinical practice, participants on the diet-trajectory are allowed to stop the diet switch to CAU after 5 weeks. This will be advised when participants do not respond to the diet (i.e. non-responders and deterioration group). Responders are allowed to add CAU to the diet, but this will not be advised as with the non-responders. Participants in the comparator arm will not be offered the possibility to switch to one of both dietary interventions to allow unbiased examination of (cost) effectiveness of the diet-trajectories.

### Procedures

Families who are interested to participate in the TRACE study are informed via telephone and information letters (sent via e-mail). Families can choose whether they want to participate in the dietary treatment arms or the CAU arm. If they choose a dietary treatment, they will be randomly assigned to either the elimination diet or the healthy diet.

If the eligibility criteria are met, parents are invited for an intake with a researcher to explain further details of participation, plan appointments and to disclose which diet the child is randomized to via a sealed envelope. Both parents fill out a written informed consent for the TRACE study (Additional file 1). If children are 12 years old, they also need to sign the consent forms. In addition, teachers are informed about the study via e-mail and general practitioners via postal letters.

**Fig. 1:**
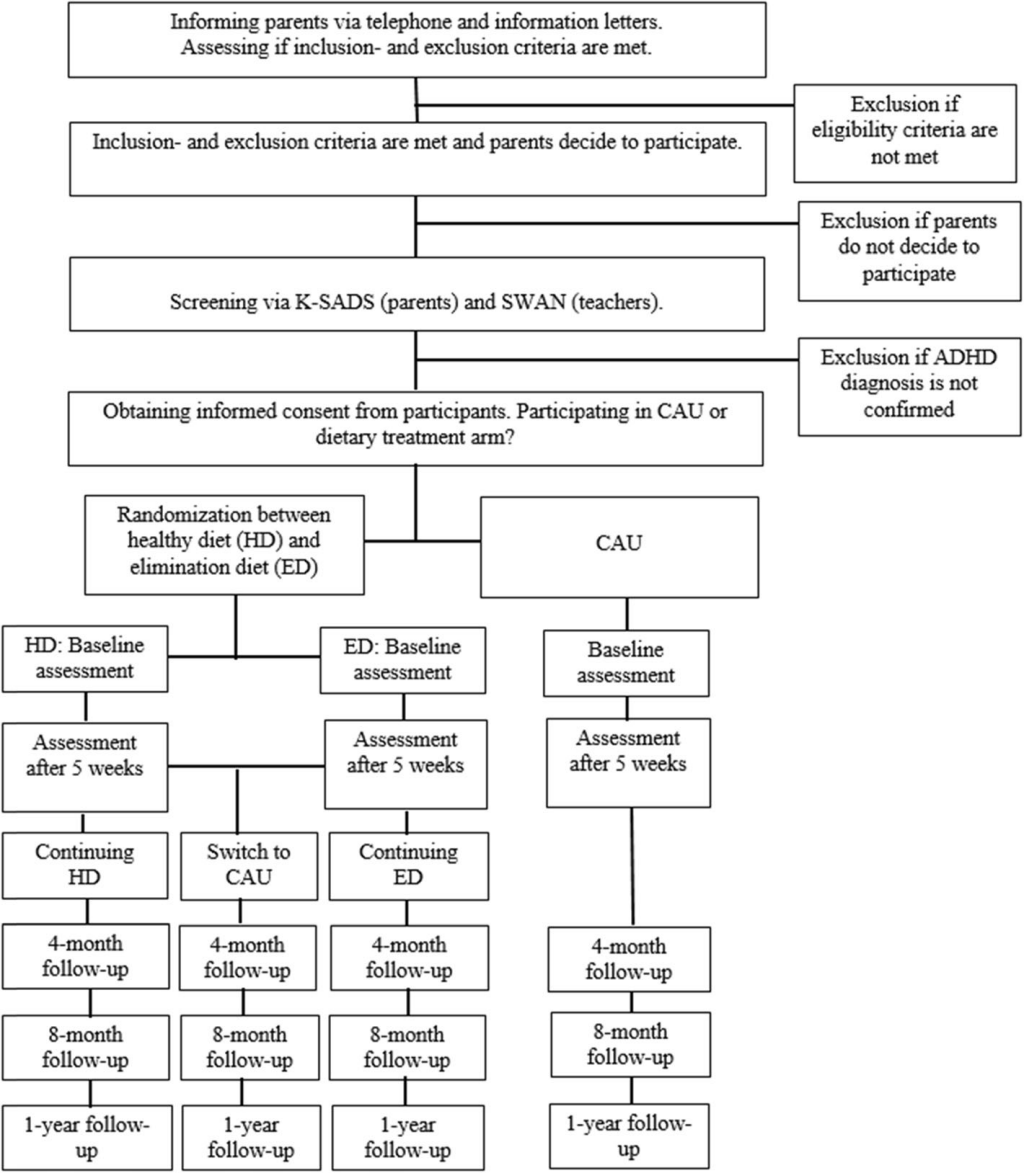
Recruitment and Procedure of the Study

Assessments will take place at baseline (T0), after five weeks of treatment (T1), after four (T2), eight (T3) and 12 (T4) months after start of treatment (i.e. dietary treatment or CAU). T0 will be scheduled within two weeks prior to the start of the treatment. Medication treatment prior to the dietary treatment has to be discontinued no later than one week before start of the diet. T0, T1 and T4 assessments will take place at one of the two participating sites. For the T2 and T3 assessments, parents are asked to fill out questionnaires online. Teachers are asked to fill out online questionnaires at T0, T1 and T4. Figure 1 depicts a flowchart on recruitment and procedure of the study.

### Interventions

In both dietary groups, weekly contacts with the dietician (via telephone or video calls) are scheduled to keep the participant and family motivated, respond to questions and assess adherence and nutritional quality. After 2 weeks, researchers contact the family to evaluate experiences with the diet so far. To keep track of daily changes in behavior, parents evaluate the severity of hyperactivity/impulsivity, attentional, physical and emotional problems on a 5-point scale (ranging from *1 no problems* to *5 severe problems*) every day (during the first 5 weeks and during the re-introduction phase of the ED). Dieticians discuss this daily assessment on the four subscales during their weekly contacts with parents. This daily assessment is specifically developed for the present study. To facilitate feasibility and adherence to the diet, parents receive examples of menus, recipes, shopping lists and advice for situations outside their home (e.g. parties).

#### Elimination diet

The goal of the Elimination Diet (ED) is to diminish symptoms of ADHD and emotion dysregulation symptoms by means of the exclusion of specific food components. The first part of the ED trajectory consists of a 5 week elimination phase where children follow a standardized ED supervised by a dietician (see Additional file 1: Appendix A). All known food allergens (proteins from milk, egg, wheat, fish (including shellfish and mollusks), soy, peanuts and nuts) and potential food triggers (gluten and histamine- releasing or histamine-containing products: see Additional file 1: Appendix A) will be eliminated. In addition, sugar intake is restricted in the elimination phase. Parents will receive a detailed list of products that children are allowed to eat during the elimination phase.

The second part (reintroduction phase) may last up to 12 months and consists of four phases (see Table 1; see Additional file 1: Appendix B). Every 14 days a new food is introduced according to a standardized scheme in a sufficient amount as to be able to trigger ADHD symptoms. In reintroduction phase one food allergens are reintroduced one by one to the standardized ED. If the reintroduction of a food allergen does not trigger recurrence of ADHD symptoms (based on the daily assessments of parents), this food allergen is added to the diet - after phase one is completed - and can be eaten again. If a food allergen does seem to trigger recurrence of ADHD symptoms (based on the daily assessments of parents), the food allergen is listed in the category ‘to be avoided’. In the next week, no new food allergen introduction takes place to allow the ADHD symptoms to decrease again to the level prior reintroduction. When ADHD symptoms have returned to the level prior reintroduction (based on the daily assessments of parents), another new food allergen is introduced in the week thereafter. Between phase one and two is a period of 2 weeks in which the standardized ED is followed, complemented with food allergens that did not trigger ADHD symptoms (‘Baseline diet +’). In the following phases sugar (phase two), histamine-releasing or histamine-containing products (phase three) and additives (phase four) are reintroduced. The procedures during reintroduction phases three and four are similar to reintroduction phase one. During phase two, accumulating amounts of sugar are added during 8 days. Despite the lack of consistent objective data demonstrating effects of sugar on cognition or behavior, subjective reports - by for example parents - of adverse effects of sugar are widespread [22, 23]. Therefore, we also assess whether sugar may trigger recurrence of ADHD symptoms.

**Table 1:** Re-introduction Phase Elimination Diet

Phase	Period	Diet
*Elimination phase*	Weeks 1–5	Baseline ED
*Re-introduction phase*		
**Phase 1: allergens**	± 3 months	Baseline ED
Subsequently: proteins from milk, egg, wheat, fish, soy, peanuts, nuts		
	2 weeks	Baseline diet +
**Phase 2: sugar**	8 days	Baseline diet +
**Phase 3: histamine-releasing or histamine-containing products**	± 2.5 months	Baseline diet +
Subsequently: aromatic substances, specific foods, biogenic amine, nitrate, cocoa		
**Phase 4: additives**	± 2.5 months	Baseline diet +
Subsequently: sorbic acid, sulphites, glutamates/glutamic acid, artificial colorings and further examination (if needed) of products that child responded to by re-introducing products again		

During the reintroduction phase, parents have contact with a dietician every 2 months to identify foods that trigger ADHD symptoms in their child, to give mental support and to answer questions about the diet. In addition, depending on the parents’ needs, the researchers can provide mental support and advice to the parents via telephone. Furthermore, all foods and/or food components that have to be avoided are registered. Eventually this phase leads to a consolidated dietary advice about the specific foods to be avoided, while maintaining an otherwise normal diet. Participants who drop-out at any time can switch to CAU.

#### Healthy diet

The healthy diet aims to balance possible deficits in nutrient intakes or excessive intakes of nutrients, in order to reduce ADHD symptoms and emotion dysregulation symptoms (see Additional file 1: Appendix C). This diet is based on the Dutch dietary guidelines 2015 and the recommended daily consumption of food groups derived from those guidelines per sex and age group, as made by The Netherlands Nutrition Center (Table 2) [24, 25].

**Table 2:** Recommended Daily Intake Food Groups

Food group	4–8 years Boys and girls	9–13 years Boys	9–13 years Girls
**Vegetables**	100–150 g 2–3 serving spoons	150–200 g 3–4 serving spoons	150–200 g 3–4 serving spoons
**Fruit**	1.5 pieces	2 pieces	2 pieces
**Bread, brown or whole-grain**	2–4 slices	5–6 slices	4–5 slices
**Grain products** (e.g. brown rice, whole-grain pasta) or potatoes	2–3 potatoes / serving spoons	4–5 potatoes / serving spoons	3–5 potatoes / serving spoons
**Dairy**	2 portions (300 ml)	3 portions (450 ml)	3 portions (450 ml)
**Cheese**	20 g 1 slice	20 g 1 slice	20 g 1 slice
**Meat, fish, chicken, eggs, vegetarian products, legumes** Variation is important!	Choose every week for example: -max. 250 g meat^a^ − 100 g fish − 2-3 eggs − 1-2 serving spoons legumes	Choose every week for example: -max. 500 g meat^a^ − 100 g fish − 2-3 eggs − 2 serving spoons legumes	Choose every week for example: -max. 500 g meat^a^ − 100 g fish − 2-3 eggs − 2 serving spoons legumes
**Nuts**	15 g	25 g	25 g
**Soft or liquid spreadable fats and cooking fats**	30 g^b^	45 g^b^	40 g^b^
**Fluid**	1–1.5 l	1–1.5 l	1–1.5 l

^a^*1 portion meat for 4–8 year olds = 50 g; for 9–13 year olds = 75 g*

^b^*5 g per slice; 15 g is 1 table spoon*

Consequently, some foods are allowed in unlimited quantities and frequencies (e.g. vegetables), others in restricted quantities and frequencies (e.g. honey), some in very restricted quantities and frequencies (e.g. processed meat) and some foods are not allowed (e.g. white bread) (see Additional file 1: Appendix C). Parents receive this detailed list of which foods are allowed in which quantity and frequency (see Additional file 1: Appendix C). This healthy diet is prescribed in a strict and structured manner (e.g. stressing the importance of adhering to the diet), making the diet comparable to ED regarding impact to the family, household structure and attention towards the child. It is not the intention to affect the allergen content of the diet or losing or gaining weight.

Contact frequency with a dietician and researchers is similar to the ED. The second phase of the healthy diet consists of two-monthly supervising by a dietician. During these supervisions, adhering to the diet and the ADHD symptoms and emotion dysregulation symptoms are evaluated (see Additional file 1: Appendix D).

#### Care as usual (CAU)

According to the Dutch Multidisciplinary guidelines for the diagnosis and treatment of ADHD [26] and authoritative international guidelines [27], CAU for elementary school–aged children (5–12 years of age) with ADHD consists of the prescription of medication approved for ADHD and/or evidence-based parent and/or teacher-administered behavior therapy, preferably both medication and behavior therapy. First-line option for medication is methylphenidate, second-line option is lisdexamfetamine or dexamfetamine, and third-line options atomoxetine or guanfacine [27]. The school environment is part of the treatment plan by instructing teachers how to (a) best modify the classroom environment to minimize distractions for the child and (b) give teaching instruction to the child with maximum learning efficiency.

### Outcomes

Outcome parameters will be assessed as shown in Table 3.

**Table 3:** Outcome Parameters from Baseline to 12 Months after Baseline

Measurement	Time^**a**^	Instrument
**Descriptives**		
Prior beliefs	T0, T1	5-item questionnaire
IQ	T0	WPPSI-III or WISC-III
Demographics	T0	Separate questions
Parental psychopathology	T0, T2, T3	GHQ
Parental ADHD symptoms	T0	ADHD Rating Scale
**Primary outcome**		
Respondership	T1, T4	SWAN, SDQ
**Secondary outcomes**		
Blinded ADHD assessment	T0, T1, T4	DB-DOS
Parent & teacher comorbidity ratings	T0, T1, T4	SDQ, CSBQ, BRIEF, UPPS-P, EDI
Cognitive performance	T0, T1, T4	COTAPP
School performance	T0, T4	Monitoring system Dutch education
Motor activity and sleep pattern	T0, T1, T4	Actigraph
Physical measurements	T0, T1, T4	Weight, height, blood pressure, heart rate
Somatic complaints	T0, T1, T2, T3, T4	Pittsburgh side-effects rating scale
Sleep problems	T0, T1, T2, T3, T4	5-item questionnaire
Quality of life	T0, T1, T2, T3, T4	EQ-5D
Food consumption & eating habits	T0	Questionnaire dietary pattern
	T0, T4	Nutritional assessment (‘Eetmeter’)
Nutritional quality	T0, T2, T3, T4	Nutritional assessment (‘Eetmeter’)^b^
	T0, T1, T2, T3, T4	Expert view
	T0, T1, T4	Blood sample
Carer-related quality of life	T0, T1, T2, T3, T4	CarerQol, PSQ
Parenting styles and family functioning	T0, T1, T4	FFQ, BSBP
Adherence	T1, T4	Morisky Adherence Scale, food diary, separate questions
Satisfaction	T1, T4	GGZ-Thermometer
Cost measurements	T1, T2, T3, T4	Tic-P

*WPPSS-III* Wechsler Preschool and Primary Scale of Intelligence, *WISC-III* Wechsler Intelligence Scale for Children, *GHQ* General Health Questionnaire, *SWAN* Strengths and Weaknesses of ADHD-symptoms and Normal-behaviors (SWAN) rating scale, *SDQ* Strength and Difficulties Questionnaire, *DB-DOS* Disruptive Behavior Diagnostic Observation Schedule, *CSBQ* Children’s Social Behavior Questionnaire, *BRIEF* Behavior Rating Inventory of Executive Function, *UPPS-P* Urgency, Premeditation, Perseverance, Sensation seeking, and Positive urgency Impulsivity scale, *EDI* Emotion Dysregulation Inventory, *COTAPP* Cognitive Task Application, *PSQ* Parenting Stress Questionnaire, *EQ-5D* EuroQol Five Dimensions Health Questionnaire, *FFQ* Family Functioning Questionnaire, *BSBP* Brief Scale of Parental Behavior, *Tic-P* Trimbos and iMTA questionnaire on Costs associated with Psychiatric illness’

^a^T0 baseline; T1 five weeks after baseline; T2 four months after baseline; T3 eight months after baseline; T4 12 months after baseline. ^b^ Only parents of healthy diet participants who continue the diet after five weeks, register food consumption at T2 and T3

#### Primary outcome

##### Respondership

Parents and teachers are invited to rate the child’s ADHD traits using the SWAN questionnaire. The SWAN consists of 18 DSM-IV-based items scored on a 7-point Likert scale ranging from 3 (*far below average*) to -3 (*far above average*) with higher scores reflecting more severe ADHD symptoms [21]. Items 1 to 9 assess symptoms of the ADHD inattentive type and items 10 to 18 assess symptoms of the ADHD hyperactive/impulsive type. The Dutch version of the SWAN questionnaire is a reliable instrument (Cronbach’s α = .87) [28, 29] and demonstrates adequate convergent and discriminant validity [28, 29]. Parents and teachers also are asked to fill out the Strengths and Difficulties Questionnaire (SDQ) to assess emotion dysregulation [30]. The SDQ Dysregulation Profile (SDQ-DP) includes fifteen items representing emotional symptoms, conduct problems and hyperactivity-inattention [31]. The items can be answered on a 3-point scale ranging from 0 (*not true*) to 2 (*definitely true*) with higher scores indicating more emotion dysregulation problems. The Dutch version of the SDQ-DP demonstrates adequate reliability (Cronbach’s α = .85) and construct and longitudinal validity [31].

To align with the evaluation of the diet after 5 weeks, response to treatment will be evaluated by assessing the change in ADHD and emotion dysregulation symptoms at T0 and T1 or T4 (i.e. SWAN inattentive scale, SWAN hyperactive/impulsive scale and SDQ dysregulation profile rated by both parent and teacher: together six scales) [32]. A 30% or more symptom decrease will be regarded as a significant response to treatment (i.e. positive value), and a 30% or more symptom increase is regarded as significant deterioration of symptoms (i.e. negative value) [9, 33]. The primary outcome variable ‘respondership’ is divided into five categories:

1. Full responder (significant response on both parent and teacher rated scales): all scales are ≥ − 20% AND at least one of three parent rated scales shows a ≥ 30% symptom reduction AND at least one of three teacher rated scales shows a ≥ 30% symptom reduction.
2. Partial responder (significant response on parent or teacher rated scale):
  - All scales are > − 30% AND at least one of three parent rated scales shows a ≥ 30% symptom reduction AND all three teacher rated scales are < 30%.
  - OR: All scales are > − 30% AND at least one of three teacher rated scales shows a ≥ 30% symptom reduction AND all three parent rated scales are < 30%.
  - OR: All scales are > − 30% AND at least one of three parent rated scales shows a symptom reduction between 20% & 30% AND at least one of three teacher rated scales shows a symptom reduction between 20% & 30%
3. Mixed responder (significant response on at least one parent rated scale and significant deterioration on at least one teacher rated scale or vice versa):
  - At least one of three parent rated scales shows a ≥ 30% symptom reduction AND at least one of three teacher rated scales shows a ≤ − 30% symptom deterioration.
  - OR: At least one of three teacher rated scales shows a ≥ 30% symptom reduction AND at least one of three parent rated scales shows a ≤ − 30% symptom deterioration.
4. Non-responder (no significant response): all six scales are > − 30 and < 30%.
5. Deterioration (significant deterioration on at least one parent or teacher rated scale): all scales are < 30% AND at least one of three parent rated scales shows a ≤ − 30% symptom deterioration OR at least one of three teacher rated scales shows a ≤ − 30% symptom deterioration.

#### Secondary outcomes

##### Blinded ADHD assessment

The blinded ADHD assessment includes a standardized observation that is carried out using the Disruptive Behavior Diagnostic Observation Schedule (DB-DOS) [34], a method for observational assessment of disruptive behavior. This observation schedule is extended for the TRACE study, in order to also observe and assess ADHD symptoms. Though the Dutch version of the DB-DOS has been developed and validated for children below age 6, it can be extended for use in children aged 6–12 year [34]. We included this promising standardized and blinded observation as secondary outcome (instead of using this blinded measure as primary outcome), because the psychometric properties of the extended version used in the present study are not known yet.

The DB-DOS is divided into three interactional contexts: two examiner contexts and one parent context. In the first examiner context, the examiner is ostensibly responsive to child behavior, the so-called Examiner Engaged context. Second, within the context of minimal support, the child is observed while working independently, with the examiner being busy doing his or her own work (Examiner Busy context). In addition, the examiner also briefly leaves the room to probe potential covert rule-breaking behaviors. The primary caregiver is involved in the Parent context. Procedures are explained to the parent before starting the tasks and parents are provided with simple worded instructions on flip cards. The different tasks, lasting approximately 4 minutes, are parallel across the modules, including frustration, internalization of rules, compliance, and social play tasks. DB-DOS codes are ratings of child behavior ranging from 0 to 3 and comprising two categories: typical (code 0 = normative behavior and 1 = normative misbehavior) and clinically concerning behavior (code 2 = of concern and 3 = atypical). Each item is rated separately for each context by a blinded clinician (blinded to treatment group and time point).

##### Parent & teacher behavioral comorbidity ratings

To assess the presence of emotional symptoms, conduct problems, peer relationship problems and social behavior, parents and teachers are asked to complete the SDQ (25 items), Children’s Social Behavior Questionnaire (CSBQ; 40 items) and Behavior Rating Inventory of Executive Function (BRIEF; 75 items). The SDQ is a time efficient instrument and the most widely used scale in research and clinical practice, to get a reliable and valid assessment of internalizing and externalizing problems and the impact thereof on family and school functioning in children aged 3–16 years old [30]. The Dutch version of the CSBQ is a well validated, reliable instrument to assess milder forms of autism spectrum symptoms [35]. Items are answered on a 3-point scale ranging from 1 (*not applicable*) to 3 (*clearly applicable*) with higher scores reflecting more autism symptoms. The Dutch version of the BRIEF is a well validated, reliable instrument to assess executive functioning problems [36]. Items are answered on a 3-point scale ranging from 1 (*never*) to 3 (*often*) with higher scores reflecting more executive functioning problems. To assess five distinct features of impulsive behavior, parents fill out the Urgency, Premeditation, Perseverance, Sensation seeking, and Positive urgency Impulsivity scale (UPPS-P) [37, 38]. Each item of the UPPS-P is rated on a 4-point Likert scale ranging from 1 (*strongly agree*) to 4 (*strongly disagree*). Finally, parents fill out the Emotion Dysregulation Inventory (EDI) which is a caregiver-report questionnaire assessing emotion dysregulation symptoms. The EDI consists of 32 items which are rated on a 5-point scale (*not at all* to *very severe*) regarding the emotion dysregulation symptoms occurring in the previous 7 days. In addition, the items are also scored on a ‘lifetime’ prevalence scale, ranging from ‘same, worse, better, or new (symptom never occurred before)’. The psychometric properties of the Dutch versions of the UPPS-P and EDI are not known yet.

##### Cognitive performance

Cognitive assessment is performed using the Cognitive Task Application (COTAPP) [39]. This Dutch computerized neurocognitive assessment tool is designed to measure (variability in) processing speed, attentional and executive control, working memory and learning speed. The COTAPP is a two-choice reaction time paradigm and consists of seven blocks, in which the child is guided through the different paradigms in a play-like manner. By default no coaching is offered to the child. If the child does not succeed in completing the task without the assistance of the examiner, coaching or help can be offered in a structured manner. The amount of offered help is coded by the examiner and included in the outcome parameters. Furthermore, the level of verbal and motor activity of the child during performance can be coded. Validity and reliability of the COTAPP have been confirmed [39], with COTAPP relating significantly to intelligence, school performance, ADHD and Autism Spectrum Disorder symptoms and quality of the student-teacher relationship.

##### School performance

School performance is evaluated on the basis of the monitoring system used in Dutch schools. This monitoring system includes a set of standardized tests measuring motor development, language development (reading, spelling, reading comprehension) and arithmetic skills. Performance level is expressed in percentiles compared to age-matched norms. With the permission of the parents, data can be easily obtained by contacting the school of the child. A change score per domain will be calculated and used to assess school performance.

##### Motor activity and sleep pattern

Motor activity and 24 h sleep/wake measurements including total sleep time (TST), sleep latency and wake after sleep onset (WASO) are objectively and non-invasively measured using actigraph recordings in the home situation (http://www.actigraphcorp.com/products/wactisleep-bt-monitor/), allowing for a participants’ activity information to be obtained in a natural setting for a prolonged and continuous period of time. Participants are asked to wear a wrist actigraph during a full week. The mean and variance of activity ratios of mutually exclusive intervals are analyzed from low-level to high-level (0.5–2.8 G) [40].

##### Physical measurements

Blood pressure, heart rate, height, weight, sleep problems and somatic complaints are routinely monitored at every visit as part of standard clinical care. Sleep problems will be examined using a 5-item questionnaire (answered with ‘yes’ or ‘no’) used in standard clinical care assessing problems with falling asleep, maintaining sleep and total amount of sleep compared to children of the same age. Somatic complaints (e.g. headache, nausea) will be assessed using the frequently used Pittsburgh side-effects rating scale (translated to Dutch) [41]. The 18 items can be answered on a 4-point scale ranging from 0 (*none*) to 3 (*severe*).

##### Quality of life

Health related quality of life (HRQL) is assessed using the EQ-5D Youth [42]. The English version is a validated generic HRQL instrument, that measures quality of life based on 5 dimensions: mobility, self-care, usual activities, pain/discomfort and anxiety/depression. Each dimension is assessed by one question with a 3-point response scale ranging from 0 (*no problems*) to 2 (*severe problems*) of which higher scores indicate greater impairment. In addition, parents are asked to rate the overall health of their child on a scale from 0 to 100. This questionnaire has been used in the Netherlands for assessments of quality of life in children with ADHD [42].

##### Food consumption and eating habits

Food consumption of the child (all treatment conditions) will be measured through an online tool (‘Eetmeter’, Dutch Nutrition Center) available at the website of the Dutch Nutrition Center or as a mobile app (free of charge). This tool has an extensive catalogue of products found in the Dutch supermarkets, including the common quantitates in which they are eaten. Users can enter per main meal (breakfast, lunch and dinner) or between main meals (snacks) the foods that are consumed. Parents are asked to report all food consumed by the child, for two weekdays and one weekend day and send this information to the research staff by email (an export function is part of the online tool). Based on the Dutch Food Composition Database (NEVO) nutrient intake can be calculated from the food consumption data [43]. In addition, parents fill out a general questionnaire (specifically developed for the present study) at baseline assessing the current eating habits, daily meal structure and family eating rules. This includes questions about whether the child has (food) allergies/ intolerances, follows a specific diet or uses dietary supplements, how often per week a child consumes breakfast, lunch and dinner and the number of snacks between the main meals. This questionnaire also assesses whether main meals are consumed on regular times each day, whether children have to follow certain rules (e.g. leaving table when finished, eating at the table) and how often children do not eat at home.

##### Nutritional quality

Nutritional adequacy of the overall diet is continuously monitored and regularly registered by the dietician during the whole study (based on the ‘Eetmeter’ and expert view). For the ED group, this is registered after phase one of the reintroduction phase and at the end of the reintroduction. For the healthy diet group, this is registered after one week, four and eight months of the diet. If necessary, dietary supplements are recommended to the children after T1. Also, a blood sample (15 ml) is drawn from children randomized to the elimination diet or healthy diet. Blood samples are fractionated into serum and subsequently stored at − 80 °C until further use. If nutritional deficits are expected in long term users of ED, it is possible to measure the level of those specific minerals or vitamins.

##### Adherence

Adherence to treatment is measured using the Morisky Adherence Scale [44]. The English version of this scale is the most validated scale for measuring non-adherence in clinical practice and distinguishes between intentional and unintentional non-adherence. The scale has been adapted in order to assess also adherence to a dietary treatment. The scale consists of eight items which parents can rate on a 5-point scale ranging from 0 (*never*) to 5 (*always*). Higher scores reflect more problems adhering to the treatment. During every weekly consult, dieticians register if children deviated from the diet, and what parents experienced as easy and challenging during the last week of the diet. In addition, dieticians and parents assess adherence on a 10-point scale (ranging from 1 *no adherence to the diet* to 10 *perfect adherence to the diet)* every week. Also, parents fill out a food diary during the first week of the diet which helps dieticians to assess adhering to the diet. Compliance to the dietary restrictions, foods that are used to replace the ‘forbidden’ foods and the use of dietary supplements (prescribed or on own initiative) are queried at the end of the study.

##### Carer-related quality of life

The CarerQol instrument (7 items) [45] and the Parenting Stress Questionnaire (34 items) [46] are aimed at measuring carer-related quality of life in informal caregivers. The CarerQol is an easy to use instrument and the Dutch version demonstrates good feasibility and construct validity [45]. Items are scored on a 3-point scale ranging from 0 (*none*) to 2 (*a lot*) with higher scores reflecting more burden experienced by the parent. The Dutch Parenting Stress Questionnaire (PSQ: English translation of ‘Opvoedingsbelasting Vragenlijst (OBVL)’) shows good internal consistency (Cronbach’s α from .89 to .91) and demonstrates construct validity [46]. Items are scored on a 4-point scale ranging from 1 (*not applicable*) to 4 (*completely applicable*) with higher scores indicating more parenting stress problems.

##### Parenting styles and family functioning

Family functioning and parenting styles are assessed using two validated Dutch questionnaires: the Family Functioning Questionnaire (FFQ: English translation of ‘Vragenlijst Gezinsfunctioneren Ouders (VGFO)’) [47] and the Brief Scale of Parental Behavior (BSBP: English translation of ‘Verkorte Schaal voor Ouderlijk Gedrag (VSOG)’) [48]. These are used to assess changes in family functioning and pedagogical style during treatment. The 28 items of the FFQ can be answered on a 4-point scale ranging from 1 (*not applicable*) to 4 (*completely applicable*) with lower scores indicating more problems in family functioning. The 25 items of the BSBP can be answered on a 5-point scale ranging from 1 (*(almost) never*) to 5 (*(almost) always*). Lower scores on two subscales indicate inadequate parenting styles, whereas higher scores on the three remaining subscales indicate inadequate parenting styles.

##### Satisfaction

Satisfaction and compliance is measured using the GGZ-Thermometer (http://www.ggznederland.nl/leden/thermometer/handleiding.html). This instrument allows Dutch institutions to examine how children and parents appreciate the care provided. Parents assess their satisfaction about the information they received about the treatment, amount of shared decision making, the team of psychologists and the result of the treatment. Also, parents assess experiences of the child about the treatment trajectory and of parents’ own treatment (if applicable). Finally, parents can rate their overall treatment trajectory on a scale of 0 to 10.

##### Cost measurements

Healthcare resources use, patient and family costs and productivity losses (absence from work of parents) as a consequence of the child’s psychiatric disorder are measured using the Dutch ‘Trimbos and iMTA questionnaire on Costs associated with Psychiatric illness’ (Tic-P questionnaire) [49]. Validity and reliability have been established [49]. The number of all health care contacts is registered with a 3 month recall period. These data are used to calculate the cost-effectiveness of the dietary conditions compared to CAU at 12 months of follow-up.

#### Descriptives

##### Prior beliefs

Parents prior beliefs about the short-term and long-term success and burden of dietary intervention and CAU are evaluated using a 5-item questionnaire in which parents rate their prior beliefs on a 5-point scale ranging from 0 (*completely disagree*) to 5 (*completely agree*). This measurement also will be used to examine successful randomization, by verifying whether the three groups differ in beliefs about the effectiveness of the intervention at baseline.

##### Intelligence quotient

Total IQ is estimated using five subtests of the Wechsler Intelligence Scale for Children (WISC-III) [50]: Vocabulary, Similarities, Block Patterns, Picture Completion and Digit Span. These subtests are known to correlate between .90–.95 with the Full Scale IQ [51].

##### Demographics

Ethnicity, family-structure and SES (composite measure based on the average of highest level of completed education of both parents and the family income) are recorded via single questions that parents fill out online.

##### Parental psychopathology

Parental psychopathology is measured using the General Health Questionnaire (12 items) (GHQ-12) [52] and the ADHD rating scale (46 items) [53], for identifying minor psychiatric disorders and ADHD in the general population, respectively. The English version of the GHQ-12 shows good internal consistency with an average Cronbach’s α of .90. Also, construct validity has been confirmed. Answers range from 0 (*never*) to 4 (*often*) with higher scores reflecting more symptoms of psychiatric disorders. The English version of the ADHD Rating Scale is a highly reliable questionnaire with adequate criterion-related validity [53]. Parents fill out 23 items concerning behavior in the last 6 months and 23 items concerning childhood behavior. Answers are scored on a 4-point scale ranging from 0 (*never*) to 3 (*very often*) with higher scores reflecting more ADHD symptoms.

#### Measurements CAU

In order to make participation in the CAU group less burdensome and thereby enhancing inclusion of CAU participants, there will be no blood collection by venipuncture. In addition, parents and teachers have to fill out fewer questionnaires compared with parents and teachers of children in the dietary treatment arm. Parents do not have to fill out the BRIEF, CarerQol, Family Functioning Questionnaire and the Brief Scale of Parental Behavior. Teachers do not have to fill out the BRIEF and CSBQ.

### Sample size and power

The justification of sample size is calculated based on the assumption of superiority: we assume that the ED is more effective than the healthy diet on the ordinal primary outcome respondership (i.e. five categories: full responder, partial responder, mixed responder, non-responder and deterioration). A clinically relevant outcome is defined as detecting twice as many full responders in the ED group than in the healthy diet group. Each dietary group includes 81 children. With this sample size and using ordinal regression, the power is 0.99 (α = 0.05, two sided test) to detect double the amount of full responders in the ED compared with the HD (Table 4: scenario 1). In addition, the power to detect one and a half times as many full responders in the ED compared with the healthy diet is 0.64 (α = 0.05, two sided test) (Table 4: scenario 2).

**Table 4:** Hypothetical Distribution of Participants for Power Calculation

	Full responder	Partial responder	Mixed responder	Non-responder	Deterioration
Scenario 1					
Elimination diet	16 (20)	45 (55)	10 (12%)	6 (8%)	4 (5%)
Healthy diet	8 (10%)	24 (30%)	21 (25%)	16 (20%)	12 (15%)
Scenario 2					
Elimination diet	12 (15%)	37 (45%)	13 (16%)	11 (14%)	8 (10%)
Healthy diet	8 (10%)	24 (30%)	21 (25%)	16 (20%)	12 (15%)

### Data collection and management

Participants will only be identifiable via a unique code assigned at the date of inclusion to anonymize all data. Non-anonymous data such as informed consents will be stored in password protected folders and in a locker. We use the online electronic data capture software CASTOR EDC to collect and store questionnaire data. We also enter and store data in CASTOR EDC which is collected during an assessment at a Karakter or Triversum - GGZ-NHN location, such as physical measurements, COTAPP observation form and outcomes of the IQ test. These data then are stored in a locker. Video-data of the DB-DOS and Actigraph data will be anonymously stored on a network attached storage. Only appointed researchers have access to this storage. Data collected by the dieticians, such as information collected during weekly appointments with parents, are stored in Microsoft Access. Nutritional assessments (of three days) will be stored in password protected folders. Researchers will check the data collected by dieticians bi-weekly for completeness and discuss this with the dieticians. After completion of the study, all data will be entered again manually to compare the entered data in CASTOR EDC and to check for mistakes.

Parents and teachers only can continue to a different questionnaire if all items are completed. If questionnaires are not filled out on time, researchers will remind parents and teachers first via e-mail (twice) and then via telephone. Researchers will check which problems parents and teachers encounter in order to help them to complete the questionnaires. To prevent drop-out at the last assessment after 12 months, researchers will explicitly mention the duration of the participation of 1 year during the intake. If parents refuse to attend the last assessment, researchers will check whether parents want to participate in other measurements (e.g. Actigraph, online questionnaires, food diary, stool sample) without visiting a Karakter or Triversum - GGZ-NHN location, in order to collect as much data as possible. Parents can declare their travel expenses up to 25 euros. Children will receive at T0, T1 and T4 a small gift worth five euros. At the last assessment, parents receive an extra reimbursement of 25 euros. Participants may withdraw from the study for any reason at any time.

### Statistical analyses

All primary analyses will be Intention To Treat. The primary outcome will be measured as an ordinal variable with five categories. Therefore, the effect of the intervention will be measured in terms of odds ratio, comparing odds for reducing ADHD symptoms and emotion dysregulation symptoms in the elimination diet group with the corresponding odds in the healthy diet group. Multiple logistic regression analyses will performed to compute the odds ratio and 95% confidence intervals, using ordinal logistic regression analysis. The CAU group will be used as a reference group.

Differences in continuous outcome measures between the dietary trajectories will be determined with analyses of ANCOVA. The follow-up value (T1, after five weeks) will be added as the dependent variable and the baseline value will be added as covariate. The two dietary groups will be compared on all outcomes at T1, using T0 scores as covariates. Clinical significance will be estimated using the partial eta squared effect. The CAU group will be used as a reference group.

In addition, a repeated measures ANOVA will be performed. First, the whole sample will be used (Intention to Treat) to determine whether adding a dietary treatment has beneficial outcomes on costs and effects of the whole treatment trajectory after 12 months (regardless of continuing the dietary treatment after 5 weeks or not). Next, per-protocol and post-hoc analyses will be performed using repeated measures ANOVA where the main outcome measures will be compared across the three conditions (i.e. elimination diet; healthy diet; CAU). Also, we will determine how many children adhered to the diet after 12 months, whether the intervention effects maintained after 12 months, which products are eliminated from the diet after 12 months and the nutritional quality of the attained diet after 12 months.

Finally, two moderator analyses will be performed: one comparing dietary responders versus non-responders (predicting respondership) and another comparing children/families adhering to a dietary treatment versus children/families that are not able to do so (predicting adherence). Both moderator analyses will be performed to compare children/families after 5 weeks and after 1 year of treatment.

For the cost-effectiveness analysis, all cost data will be accumulated over the 12 months period. Cost differences between the two dietary conditions and CAU will be compared. Incremental cost-effectiveness ratios will be calculated by dividing cost differences between treatments by differences in Quality Adjusted Life Years, as estimated from EQ-5D scores. Uncertainty analysis using 5000 bootstraps will be performed and cost-effectiveness planes and cost-effectiveness acceptability curves will be plotted.

The impact of missing data on the outcome measurements will be evaluated using different methods, such as Last Observation Carry Forward (LOCF) and Multiple Imputation (MI).

### Data monitoring

Adverse events (AE) are defined as any undesirable experience occurring to a subject during the study, whether or not considered related to undergoing a dietary treatment. All adverse events observed by the researchers or reported by the participants will be recorded. A serious adverse event (SAE) is an event that 1) results in death; 2) is life threatening (at the time of the event); 3) requires hospitalization or prolongation of existing inpatients’ hospitalization; 4) results in persistent or significant disability or incapacity; 5) is a congenital anomaly or birth defect; 6) may jeopardize the participant or may require an intervention to prevent one of the outcomes listed previously. All SAEs will be communicated to the coordinating investigator (Dr. Rommelse), who will be responsible for reporting this through the web portal ToetsingOnline to the accredited METC (IRB) that approved the protocol. Reporting of SAEs that are life threatening or result in death, will be reported not later than 7 days after first knowledge of the SAEs in a preliminary report. The final report will be completed no later than 8 days after the preliminary report. All AEs will be monitored until they are diminished or until they reached a stable situation. Follow up may require: 1) medical procedures; 2) additional tests; 3) referral to a general physician or medical specialist. SAEs need to be reported till end of study within the Netherlands, as defined in the protocol.

A safety review will be performed by the safety review board (SRB), with the aim to safeguard the interests of trial participants, assess the safety and efficacy of the interventions during the trial, and monitor the overall conduct of the clinical trial. The members of SRB receive and review the progress and accruing data of this trial and provide advice on the conduct of the trial to the Trial Steering Committee. The SRB informs the Chair of the steering committee if, in their view: (i) the results are likely to convince a broad range of clinicians, including those supporting the trial and the general clinical community, that one trial arm is clearly indicated or contraindicated, and there is a reasonable expectation that this new evidence would materially influence patient management; or (ii) it becomes evident that no clear outcome will be obtained.

Specific roles of the SRB: Interim review of the trial’s progress including updated figures on recruitment, main outcome and safety data.

Specific aspects:

- monitor recruitment figures and losses to follow-up
- monitoring evidence for treatment differences in the main efficacy outcome measure
- decide whether to recommend that the trial continues to recruit participants or whether recruitment should be terminated either for everyone or for some treatment groups and/or some participant subgroups

The SRB meets before the trial starts, to discuss the protocol, the trial, any analysis plan, future meetings, and to have the opportunity to clarify any aspects with the principal investigators. The SRB will meet at least yearly, interim analyses will be conducted when 50% of the participants has completed the first 5 weeks of the study. Interim analyses will include the percentage of responders in each dietary treatment and adverse effects reported. Stopping rule: when only 5% (instead of 42%) of participants are regarded responders to the dietary treatments after having included 50% of the participants, we will terminate the study. It is then unlikely that the dietary treatment-trajectory will be as effective and cost-effective as CAU. At the end of the trial there will be a meeting to allow the SRB to discuss the final data with the principal trial investigators and give advice about data interpretation.

Composition: Dr. Jos Draaisma, pediatrician at the RadboudUMC. Role: head of safety review board. Dr. Rogier Donders, statistician at the RadboudUMC. Role: statistical analyses. Dr. Helen Klip, senior researcher at Karakter Child and Adolescent Psychiatry. Role: statistical analyses.

## Discussion

The goal of the present paper was to describe the rationale, study design and methods of the TRACE study: an ongoing two arm randomized controlled trial comparing the short and long term effects of an elimination diet and a healthy diet in children with ADHD. A comparator arm including children being treated with Care as Usual (CAU) is used to compare the effects found in both dietary groups. Results from this study are expected to offer insight into the short-term (i.e. five weeks) and long-term (i.e. one year) effectiveness in reducing ADHD and emotion dysregulation symptoms and measures related to ADHD (e.g. cognitive outcomes) of an elimination diet compared with an active control group (healthy diet), possible moderators of the response to dietary treatment, nutritional quality of the attained diet and cost-effectiveness of the dietary treatments compared with CAU.

So far, several studies examined the effects of food on ADHD symptoms [11–13]. However, studies examining the effects of dietary treatments in children with ADHD suffered from several limitations (e.g. long-term effects are unknown). The TRACE study aims to fill these knowledge gaps and to take into account limitations of previous studies.

It is estimated that recruitment for the trial (*N* = 81 in both dietary groups; *N* = 60 in the comparator arm) will be completed in December 2020. Data collection for the follow-up measurements is anticipated to be completed by December 2021.

## Supplementary information

**Additional file 1.** ‘Appendices TRACE protocol paper’ including: Appendix A: Background information on the TRACE Elimination diet for dieticians and researchers. Appendix B: Background on reintroduction for the dietician and researchers. Appendix C: Background information on the TRACE balanced diet for researchers and dieticians. Appendix D: Information for parents of children who continue the diet after five weeks. Appendix E: Informed consent TRACE. Appendix F: included as mentioned in the SPIRIT checklist 33 ‘Biological specimens’. ‘TRACE protocol paper’: this includes Table 1 which should be inserted in Appendix C. This is included as an additional file, because this is a landscape table. ‘TRACE protocol paper’: this includes Table 1 which should be inserted in Appendix C. This is included as an additional file, because this is a landscape table.

**Additional file 2.** ‘SPIRIT-Checklist_TRACE_Study’.

## Data Availability

Data sharing is not applicable to this article as no datasets were generated or analyzed during the current study. The final dataset will be available (anonymized) for other researchers at the end of the study. We will assess if the aim of using the dataset is in conflict with our planned future publications. If not, the dataset can be shared with other researchers.

## Abbreviations

ADHD: Attention-Deficit/Hyperactivity Disorder
AE: Adverse Event
BRIEF: Behavior Rating Inventory of Executive Function
BSBP: Brief Scale of Parental Behavior
CAU: Care As Usual
CCMO: Committee on Research involving Human Subjects
COTAPP: Cognitive Task Application
CSBQ: Children’s Social Behavior Questionnaire
DB-DOS: Disruptive Behavior Diagnostic Observation Schedule
DSM-5: Diagnostic and Statistical Manual of Mental Disorders, Fifth Edition
DSM-IV: Diagnostic and Statistical Manual of Mental Disorders, Fourth Edition
ED: Elimination Diet
EDI: Emotion Dysregulation Inventory
EQ-5D: EuroQol Five Dimensions Health Questionnaire
FFQ: Family Functioning Questionnaire
GHQ: General Health Questionnaire
HRQL: Health Related Quality of Life
IRB: Institutional Review Board
K-SADS: Kiddie Schedule for Affective Disorders and Schizophrenia
LOCF: Last Observation Carry Forward
MI: Multiple Imputations
NEVO: Dutch Food Composition Database
ODD: Oppositional Defiant Disorder
RCT: Randomized Controlled Trial
SAE: Serious Adverse Event
SDQ: Strength and Difficulties Questionnaire
SDQ-DP: Strength and Difficulties Questionnaire – Dysregulation Profile
SES: Socioeconomic Status
SRB: Safety Review Board
SWAN: Strengths and Weaknesses of ADHD-symptoms and Normal-behaviors rating scale
TRACE: Treatment of ADHD with Care-as-usual versus an Elimination Diet
Tic-P: Trimbos and iMTA questionnaire on Costs associated with Psychiatric illness’
TST: Total Sleep Time
UPPS-P: Urgency, Premeditation, Perseverance, Sensation seeking, and Positive urgency Impulsivity scale
WASO: Wake After Sleep Onset
WHO: World Health Organization
WMO: Medical Research Involving Human Subjects Act
WPPSI-III: Wechsler Preschool and Primary Scale of Intelligence
WISC-III: Wechsler Intelligence Scale for Children
