# Supplementary material for: A two arm randomized controlled trial comparing the short and long term effects of an elimination diet and a healthy diet in children with ADHD (TRACE study). Rationale, study design and methods
Source: BMC Psychiatry. 2020 May 27;20:262. doi: 10.1186/s12888-020-02576-2 (PMC7251686; doi:10.1186/s12888-020-02576-2)
Supplement: Supplementary file 1 — Additional file 1. ‘Appendices TRACE protocol paper’ including: Appendix A: Background information on the TRACE Elimination diet for dieticians and researchers. Appendix B: Background on reintroduction for the dietician and researchers. Appendix C: Background information on the TRACE balanced diet for researchers and dieticians. Appendix D: Information for parents of children who continue the diet after five weeks. Appendix E: Informed consent TRACE. Appendix F: included as mentioned in the SPIRIT checklist 33 ‘Biological specimens’. ‘TRACE protocol paper’: this includes Table 1 which should be inserted in Appendix C. This is included as an additional file, because this is a landscape table. ‘TRACE protocol paper’: this includes Table 1 which should be inserted in Appendix C. This is included as an additional file, because this is a landscape table. [file 12888_2020_2576_MOESM1_ESM.docx]

**Appendix A**

**Background information on the TRACE Elimination diet for dieticians and researchers**

1. **Objective of the TRACE Elimination diet**

The objective of the elimination diet is to omit certain food allergens and to reduce foods containing/eliciting histamines as much as possible, thereby decreasing/eliminating the characteristics of ADHD (hyperactivity, impulsiveness and attention problems). Certain foods may be eaten in unlimited quantities, while others may be eaten in limited quantities and yet others only to a highly restricted extent or not at all. The assumed mechanisms of action are the reduction of triggers for ADHD behavior and an increase in the level of structure within the family. The difference from the healthy diet is that specific allergens are eliminated from the nutritional pattern. Neither diet is intended to lead to weight loss and/or stagnation in growth for the child.

The elimination diet begins with a five-week elimination phase, in which the ingredients listed in Table 1 are eliminated as much as possible. This extensive list of ingredients should NOT be provided to the parents. This would raise too many questions on the part of the parents and make the diet unnecessarily complicated, which would not promote compliance. Practice has shown that it works better to provide parents with a “positive” list of foods that are allowed. Moreover, the plan is for only five weeks. After the reintroduction phase, if the nutritional ingredients to which the child is over-sensitive have been identified, the parents will obviously receive a list of foods that their child should not eat, and they will be instructed on how to read labels.

To optimize the feasibility of and compliance with the diet, several concessions have been made (e.g., making sandwiches with pre-packaged cold cuts containing preservatives instead of chicken breast prepared at home). If parents know that their children should not have any preservatives and pre-packaged cold cuts are included on the list of permissible foods, this can be very confusing to them. This is another reason for not providing parents with a list of “forbidden” food ingredients.

If a child responds well to the diet (i.e., showing significant improvement in ADHD symptoms), the allergens/triggers listed below will be added to the diet one at a time throughout the reintroduction phase.

**Important:** After Week 1, ascertain whether there is a risk of nutritional deficiency (based on the eating diary), and enter this into the record in Access. This is the minimal registration. If you identify a risk of nutritional deficiency during any other moment of contact, this should be registered as well.

*Table 1. Overview of allergens/triggers that will be eliminated in the elimination diet (and why) throughout the elimination phase (weeks 1–5) in the TRACE study.*

| **Nutritional ingredient** | **Reason for elimination** | **Comment** |
| --- | --- | --- |
| Milk | Allergen |  |
| Egg | Allergen |  |
| Wheat* | Allergen |  |
| Gluten* |  | Not evidence-based, but best practice. Do eliminate. |
| Fish (including shellfish and mollusks) | Allergen |  |
| Peanuts | Allergen |  |
| Nuts | Allergen |  |
| Soy (liquid/fermented) | Allergen | We do not want to replace regular milk with soy milk (a potential risk product), thereby drastically increasing soy consumption compared to what children normally use. Soy flour in products is permitted in limited quantities. Very few people have soy allergies. Soy in plant-based margarine is soy lecithin (this is permitted). |
| Foods containing/releasing histamines | Trigger; non-allergic food-sensitivity reaction | The complete elimination of histamines from the diet is not possible. For this reason, certain foods that contain/release histamines are allowed within the TRACE elimination diet (e.g., pre-packaged cold cuts). This is intended to increase feasibility and compliance. In addition, only small amounts are eaten (cold cuts no more than twice a day).  An overview of foods and additives that could result in the release of additional histamines in the body is provided on pages 2 and 3. |

*Corn, buckwheat, and rice are allowed.

There are some foods that we are not going to eliminate:

- Sugars (no evidence). We will nevertheless normalize them.
- Chicken, beef

Table 2 presents an overview of **foods and additives** that are known to contain histamines or that could increase the release of histamines in the body. This is not a diet list for parents/children. It is provided as informative, general background information intended particularly for the dietician. The food allergens that are eliminated in the TRACE elimination diet are listed here. The overview is primarily useful and important during the reintroduction phase. This list has therefore not been incorporated into table 1.

*Table 2. Overview of foods and additives that are known to contain histamines or that could increase the release of histamines in the body.*

| **Foods** | |
| --- | --- |
| Cheese | all types, except for fresh (non-aged) cheese: quark, cottage cheese, fresh cream cheese and Dutch processed cheese and cheese spreads |
| Meat | pork, pork products (e.g., ham, sausage, dry sausage varieties), meat extracts, smoked products—including smoked meat, smoked sausage, chicken livers) |
| Game | wild game |
| Fish | anchovies, herring, smoked mackerel, pilchards, sardines, haddock liver, tuna |
| Vegetables | eggplant, spinach, tomatoes, sauerkraut, white mushrooms and other mushrooms |
| Fruit | pineapple, banana, strawberries, citrus fruits (lemon, grapefruit, kumquats, lime, limequats, tangerines, mineolas, pomelo, oranges, tangelos, Ugli fruit) |
| Nuts and peanuts | particularly walnuts, pecans. To a lesser extent, other nuts and peanuts |
| Miscellaneous | brewer’s yeast, yeast extract/Marmite®, soy, soy sauce, tempeh, tamari, shrimp paste, cocoa, caffeine, egg white, herbs and spices, many aromas/essences, licorice, boxed/packaged mixes (see Additives) |
| **Additives** | |
| AZO dyes | *E102: tartrazine, E110: orange yellow S, E122: AZO Rubine, E123: amaranth, E120: cochineal, E124: cochineal red A, E128: Red 2 G, E129: Allura red A C, E150b/d: Sulphite caramel, E151: Brilliant Black BN, E154: Brown FK, E155: Brown HT, E180: Lithol Rubine BK.*  Used in products including candy, soft-drink mixes, sweet spreads, pudding, fruit sauces |
| Preservatives | *sorbic acid and sorbates (E200–E203).*  Used in products including margarine, light margarine, sweet spreads, canned vegetables, soft-drink syrup, rye bread, yoghurt with fruit |
|  | *benzoic acids and benzoates (E210–E219), preservative*.  Used in products including pre-packaged sauces, soup, soft-drink syrup, cookies, sweet spreads, fruit purées/sauce/yoghurt |
|  | *sulphites (E220–228), preservative).*  Used in products including potato products, canned fruit and vegetables, alcoholic beverages, sauces, soups |
|  | *nitrites (E249 and E250), preservative.*  Used in cheese, meat and cold cuts |
|  | *nitrates (E251 and E252) preservative*  Used in cheese, meat and cold cuts  Nitrates are found in varying quantities in vegetables. |
| Flavor enhancer | *glutamic acid/glutamate (E620–625)*  Used in products including pre-packaged soup, sauces, powdered gravy mix, mixed spices—like chicken spices or fish spices—aromas, MSG, salty snacks, pre-packaged meat, chicken or fish dishes, cold cuts |
| Scents and flavorings | *vanilla and vanillin*  Used in products including jam, cocoa powder, vanilla sugar, bonbons, chocolate products, chocolate milk, ice cream, light margarine, pudding mix, pre-packaged desserts, chewing gum, soft-drink syrups, imitation whipped cream, liqueurs, breakfast cereals, breakfast cake, cookies and cakes, muesli |
|  | *cinnamon and cinnamaldehyde*  Used in products including mixed herbs and spices, soft drinks (e.g., coke), ice cream, candy, bonbons, cough drops, chewing gum, bakery products, meat products, vermouth, liqueurs, herbal beverages |

**Appendix B**

**Background on reintroduction for the dietician and researchers**

The reintroduction phase consists of four sub-phases (Table 1). A new product is introduced every two weeks. The product should be used for 10, 7, or 5 consecutive days, depending on the sub-phase.

Reintroduction occurs in bulk (not in gradually increasing quantities). A potential tolerance limit can be investigated later. During the first sub-phase, allergens are reintroduced one at a time. If parents would like to use an order that differs from the one included in the scheme, this could be possible, in consultation with the dietician. In the second sub-phase, sugar is reintroduced and in the third and fourth sub-phase the triggers (foods that cause the release of histamines in sub-phase three and additives in sub-phase four).

If complaints occur, the use of the product should be stopped immediately. The product cannot be reintroduced until all of the complaints have disappeared. In practice, however, it is highly unlikely that this will prevent the introduction of the next product according to the scheme.

Parents should note any complaints and the nature of the reaction, along with the type and amount of the product used on a registration form (available in Dropbox). On Day 1, the parents start registering the extent of complaints on the complaint-registration form and, on Day 2, the new food product is started.

If a child truly does not like an allergen and has therefore not eaten it very often (this applies specifically to soy and fish), it does not need to be reintroduced.

During the first sub-phase, children must adhere to the basic elimination diet that was used during the elimination phase. Once all of the allergens have been introduced, those that have not caused any reactions may be added in the second and third sub-phases of the reintroduction (i.e., a Basic diet +). There is a two-week break between the first and second phase. The second sub-phase may be started once the child has followed the basic diet, supplemented with the foods that were tolerated well, for two weeks.

If a child does not like eggs and has skipped the reintroduction of eggs, the reintroduction can now be carried out. This can be done in combination with allergens that have not caused a reaction (e.g., in the form of cake or cookies containing egg).

The reintroduction of additives and substances leading to the release of histamines (Sub-phases 3 and 4) is done in groups.

If a child reacts to a group, continue to introduce the next group (AND ALWAYS RETURN TO THE BASIC + DIET!), until all groups have had a turn. Thereafter, try to identify the cause within a group to which the child reacts. Only after this has been done well can the child return to eating everything (obviously with the exception of the triggers/allergens to which the child reacts). If the groups that cause complaints have been identified after the reintroduction phase, an adjusted diet can be applied.

*General comment:*

*In some cases, a child who has had a reaction may be returned to the baseline sooner. This will allow the reintroduction to be continued sooner. Be sure to maintain a week. Example: After two days of reaction, the food is discontinued. The reintroduction can be continued after seven days instead of 12 days.*

*Table 1: Schematic overview of the reintroduction phase*

| **Phase** | **Diet** | **Period** | **Food groups** |
| --- | --- | --- | --- |
| *Elimination phase* | Baseline ED | Weeks 1-5 |  |
| *Re-introduction phase* |  |  |  |
| Phase 1 |  | ± 3 months | *Allergens* |
| 1 | Baseline ED |  | Proteins from milk |
| 2 | Baseline ED |  | Egg |
| 3 | Baseline ED |  | Wheat |
| 4 | Baseline ED |  | Fish |
| 5 | Baseline ED |  | Soy |
| 6 | Baseline ED |  | Peanuts |
| 7 | Baseline ED |  | Nuts |
| Baseline diet + |  | 2 weeks |  |
| Phase 2 |  |  | *Sugar* |
| 8 | Baseline diet + | 8 days |  |
| Phase 3 |  | ± 2.5 months |  |
| 9 | Baseline diet + |  | Aromatic substances |
| 10 | Baseline diet + |  | Specific foods |
| 11 | Baseline diet + |  | Biogenic amine |
| 12 | Baseline diet + |  | Nitrate |
| 13 | Baseline diet + |  | Cocoa |
| Phase 4 | Baseline diet + | ± 2.5 months | *Additives* |
| 14 | Baseline diet + |  | Sorbic acid |
| 15 | Baseline diet + |  | Sulphites |
| 16 | Baseline diet + |  | Glutamates / glutamic acid |
| 17 | Baseline diet + |  | Artificial colorings |
| 18 onwards | Baseline diet + |  | Further examination of products that child responded to by re-introducing products again |

**Important:** After the reintroduction of allergens (approx. 3–4 months), ascertain whether there is a risk of nutritional deficiency (based on the Eating Monitor), and enter this into the record in Access. Do this again upon the conclusion of the entire reintroduction. This is the minimal registration. If you identify a risk of nutritional deficiency during any other moment of contact, this should be registered as well.

**Appendix C**

**Background information on the TRACE balanced diet for researchers and dieticians**

1. **Objective of the TRACE balanced diet**

The objective of the balanced diet is to adjust the existing eating pattern toward a normalized nutritional pattern, thereby decreasing/eliminating the characteristics of ADHD (hyperactivity, impulsiveness and attention problems). The underlying principle in this regard is the recommended amount of foods for boys and girls, adjusted to the age of the child and a healthy product choice, as published by the Voedingscentrum (Netherlands Nutrition Centre). Certain foods may be eaten in unlimited quantities, while others may be eaten in limited quantities and yet others only to a highly restricted extent or, ideally, not at all. The assumed mechanisms of action for the normalization of the nutrition pattern are an increase in the level of structure within the family and the optimization of the intake of nutrients. Many children have an excessively one-sided nutrition pattern and consume insufficient amounts of certain vitamins and minerals and/or excessive amounts of sugar and fat. The scientific literature contains indications that such nutritional patterns can have an effect on ADHD symptoms. The difference from the first diet is that no specific allergens/triggers are eliminated from the nutritional pattern. Neither diet is intended to lead to weight loss and/or stagnation in growth for the child.

1. **Dietary recommendations for a balanced diet**
2. Eat according to the recommended amount of foods (Netherlands Nutrition Centre)
3. Have three main meals and 2–4 snacks each day.
4. Certain foods are allowed, and others may be eaten in limited amounts or ideally not at all.
   1. **Which foods are allowed?**

The choice of which foods are allowed, and which may be eaten in limited amounts or ideally not at is all derived from the Netherlands Nutrition Centre’s recommendations for a healthy product choice. In general, the products in the food pyramid are allowed without restriction. It is important to note that “allowed without restriction” does not mean that they are allowed in unlimited quantities. Quantities should be determined taking into account the recommended amounts of foods for children (see the table in the general information for parents). In general, the products outside the food pyramid are allowed in limited (or highly limited) amounts. An overview of foods that are allowed and those that are allowed with restrictions and those that are not allowed is provided in Table 1.

**Important:** After Week 1, ascertain whether there is a risk of nutritional deficiency (based on the eating diary), and enter this into the record in Access. If parents continue the diet after five weeks: ascertain whether there is a risk of nutritional deficiency (based on the Eating Monitor) after 4–8 months and enter this into the record in Access. This is the minimal registration. If you identify a risk of nutritional deficiency during any other moment of contact, this should be registered as well.

[Insert Table 1 here: see ‘Additional file 1’]

**Appendix D**

**Information for parents of children who continue the diet after five weeks**

Dear parent(s)/guardian(s),

Your child has participated in the TRACE study, which is aimed at examining whether ADHD symptoms in children can be decreased through nutrition. The results indicate that your child’s ADHD symptoms decreased due to a diet based on the food-choice guidelines of the Voedingscentrum (Dutch Nutrition Centre). You have chosen to continue adhering to these guidelines.

You and your child can continue with the recommendations that you have received. For one year, we will contact you every two months (i.e., six times) to schedule an appointment with the dietician. During these appointments, we will evaluate how things are going with following the food-choice guidelines and with your child’s ADHD symptoms. If you have questions between appointments, please contact your dietician. The information is provided on the cover of this document.

Prior to the second, fourth, and sixth consultations (i.e., after 4, 8, and 12 months), you will be asked to record your child’s food intake for three days (two weekdays and one weekend day). To this end, you can use the Voedingcentrum’s Eating Monitor, just as you did at the beginning of the study. We will also ask you to complete the questionnaire on characteristics of ADHD and complaints in children. Please send the completed information to your dietician by email within one week preceding the appointment with the dietician. As a reminder of this appointment, the dietician will send you an email containing the relevant questionnaire and instructions for completing the Eating Monitor.

**Nutritional recommendations for your child**

- Continue to use the table indicating the foods in each food group that are allowed daily and those that are allowed with restrictions (e.g., as a daily or weekly choice).
- Use the recommended amounts of foods that are appropriate to your child’s age. Children who eat in this way will consume sufficient nutritional substances. It is important to realize that these recommendations are based on averages. Slightly more or slightly less might be better for your child.
- Give your child 2–4 between-meal snacks, spread throughout the day. This can be done mid-morning, in the afternoon, and possibly after the evening meal.
- If your child has tinted or dark skin, give 10 micrograms of Vitamin D each day. This can be done in the form of either drops or tablets.
- Tell people at school, the parents of your child’s friends, relatives, and acquaintances that your child is benefiting from nutrition according to the food-choice guidelines. A lunchbox containing recommended snacks can be helpful in this regard.

*Additional tips and suggestions are available at the website of the Voedingscentrum (www.voedingscentrum.nl). Supplementary information is provided under the ‘My Child’ tab.*

**Appendix E**

*Informed consent TRACE*

Name of child:

Date of birth:

| I have read the information about the TRACE study with care. I fully understood and agree. | 0 yes |
| --- | --- |
| I was given the opportunity to process the information and to ask questions. A TRACE team-member has answered my questions satisfactorily. | 0 yes |
| I am fully aware that participation in this study is voluntarily and that I can withdraw my consent to participate in this study at any time. I also have the right to cancel my permission to use and disclose further information collected about my child at any time. I am aware that this has no influence on our received care or rights. Travel expenses will be granted with regard to the moment of withdrawal. | 0 yes |
| I give permission to inform my child his/her general practice doctor about participating in this study. | 0 yes 0 no |
| I am aware that authorized members of the Dutch Healthcare Authority could peruse medical records of my child and the data that is collected for this study. I allow these members to peruse this medical records. | 0 yes |
| I am fully aware what participation in this study means. | 0 yes |
| I agree with fulfilling online surveys. | 0 yes |
| I agree with participation in a clinical interview regarding my child. | 0 yes |
| I agree with the members of this study observing my child his/her behavior. | 0 yes |
| I agree with the members of this study measuring my child his/her intelligence and cognitive ability. | 0 yes |
| I agree with the members of this study tracking my child his/her activity with an activity tracker. | 0 yes 0 no |
| I give permission to draw my child his/her blood at three times during this study. | 0 yes 0 no |
| I agree with the storage of my child his/her bloodsamples for 10 years after particiaption in this study. | 0 yes 0 no |
| I am aware that all information about my child his/her participation will be kept anonymous. | 0 yes |
| I agree with sharing my child his/her behavioral observation (video) with other researchers on behalf of an objective evaluation of the behavior observed. | 0 yes |
| I give permission to use of my child his/her anonymous data for scientific purposes other than the TRACE study. | 0 yes 0 no |
| I give permission to possibly contact me again for follow-up research. | 0 yes 0 no |

The informed consent needs to be fulfilled before start of the study, by both the parents or legal caretakers of the child.

Name:…………………………………..... Date of birth:………………………… (dd/mm/yyyy)

Signature:…………………………. Date and place:………………………..

Name:…………………………………..... Date of birth:………………………… (dd/mm/yyyy)

Signature:…………………………. Date and place:………………………..

**Statement by the researcher taking consent:**
I have accurately read out the information sheet to the parent of the potential participant, and to the best of my ability made sure that the person understands our research. I guarantee a protection of participants privacy following the Dutch laws.

Name:……………………………… Signature:…………………………………

**Appendix F**

*TRACE-BIOME*

If parents agreed for also participating in the TRACE-BIOME study, the following material will be collected: stool (microbiome, fatty acids), saliva (hormones), and blood (fatty acids, immunoglobulin, cytokine, genotype). Blood collection by venipuncture already takes place on each of the main assessment time-points (T0, T1, T4) in the TRACE-study in order to determine whether there are potential insufficiencies in nutrients levels. Thus, there is no additional venipuncture needed for blood collection for the TRACE-BIOME study; an additional volume of 20 ml (at T0) or 10 ml (at T1 and T4) maximally are collected in this study.

**Microbiome and fatty acids.** Intestinal microbiome composition will be determined from self-collected stool samples. The stool samples are collected at home in specially designed containers (that stabilize the feces and allows for up to 14 days of storage at room temperature) and then sent by post to the research facility. Participants need to send the sample back within 7 days of collection (in order to avoid unnecessary delays in processing the stool samples associated with delayed shipping or weekend delivery). The stool samples are aliquoted and immediately frozen at -80 °C until further analyses of the short-chained fatty acids from fecal matter (34). We will use the Quantitative Insights Into Microbial Ecology (QIIME) tool to perform microbial community analysis.

**Hormone levels.** Saliva samples are taken to measure hormonal concentrations. A standard plastic tube will be used to collect 1.5 ml of saliva for determination of (stress) hormone levels, such as cortisol. Only if participants are not willing to provide blood for genotyping, saliva samples for (epi)genetics will be collected into Oragene-DISCOVER OGR-500 and Oragene RE-100 saliva tubes (4 ml in total). Saliva samples are stored at a temperature of -80°C to preserve hormone (and RNA) levels. The time of collection is registered to minimize differences in hormone concentration between participants due to day/night rhythm.

**Immunoprofiling and genotype (DNA).** A trained nurse draws blood by means of venipuncture to allow for determination of nutrients intake. For the TRACE-BIOME, additional blood is taken consequently for assessments of levels of short-chained fatty acids, immunoglobulin, proinflammatory cytokines, and for isolation of DNA (only at baseline) and RNA. Blood is collected in separate tubes that are specific for each of the parameters. Plasma is separated from cells by centrifugation if necessary and stored at -80°C. Only if participants are not willing to provide blood for genotyping, saliva samples for (epi)genetics will be collected (see above: hormone levels). DNA will be isolated using standard techniques. It will be analyzed for common and rare genetic variants of candidate genes involved in ADHD or associated gene/ neurotransmitter systems and gene pathways. The extracted DNA samples from peripheral blood are also suitable for epigenomic profiling. The microRNA (miRNA) levels of blood-expressed ADHD-relevant genes will be monitored. Candidate miRNA and mRNA serum levels will be quantified.

*TRACE-MRI*

A subsample of the TRACE participants (N=30 elimination diet and N=30 healthy diet) will be asked to participate in the TRACE-MRI study.

**Preparation for the MRI session.** Before the MRI session, children will undergo a simulation procedure to become familiar with the scanner environment and to practice the fMRI tasks. The dummy scanner is a replica of an MRI scanner and mirrors all relevant aspects, such as speakers for simulating sounds and a table for simulating experiences the participant will have in the real scanner. Pre‐recorded scanner sounds will be played. Only after acclimating the participant to the scanner environment, so that both the child and parent/guardian are comfortable with the procedure, will the participant be taken to the real scanner. If the child or his parent/guardian is uncomfortable with any aspect of the procedure, the study will be terminated. At the beginning and end of the MRI preparation, a Visual Analogue Scale ranging from 1 to 10 will be administered, to assess how anxious the participant is about the MRI session. If anxiousness is rated 8 or higher after the preparation, the MRI session will be cancelled.

**MRI acquisition.** Participants will be asked to remove all metal objects before entering the scanner room. Special care will be taken to make the child comfortable on the scanner bed. Attention will be paid to the even distribution of head support to avoid stiffness or local pressure and thereby reducing the chance of excessive motion. Participants will be provided with earplugs to protect against scanner noise. Once in the MRI scanner, the participant can see the area near the feet through a 45‐degree angle mirror fixed to the head coil. The display is connected to a projector, which projects onto a screen in the scanner room. Both the PC and the projector are located in the control room. The participant will be able to communicate with the experimenter over an intercom and can press an alarm button in case of emergency. The MRI session consists of different types of scans that range between 5 to 15 minutes, with short breaks in between, and takes at maximum 50 minutes.

We will assess the following MRI measures:

- Anatomical MRI scan (sMRI)
- Resting state MRI scan (rsMRI)
- Task‐based functional MRI scans (fMRI)
- Magnetic Resonance Spectroscopy (MRS)

**Anatomical scan.** We will acquire a high‐resolution MRI scan that is optimized for volumetric measurement of individual brain areas and of grey and white matter volumes (duration 5 minutes).

**Resting state scan.** To assess intrinsic functional connectivity between different grey matter brain regions, fluctuations in the Blood‐Oxygen‐Level‐Dependent (BOLD) signal will be measured while the participant is not performing a task (eyes open, 9 minutes).

**Task fMRI scans.** BOLD fMRI is sensitive to the concentration of deoxygenated haemoglobin in local tissue, and is now widely used to measure neural metabolism induced by task manipulations. Two sets of fMRI scans will be made to assess BOLD responses when subjects are performing tasks probing emotional matching and inhibition.

- The emotional faces task is a matching paradigm including angry, fearful, happy and neutral emotional expressions as well as a low‐level baseline (Hariri, Bookheimer, & Mazziotta, 2000). Participants have to match a stimuli presented at the top of the screen to one of two stimuli presented at the bottom of the screen, based on emotion (experimental conditions) or shape (control condition) (duration 8 minutes).
- During the stop signal task (Rubia, Halari, Mohammad, Taylor, & Brammer, 2011) participants respond to the direction of an arrow. Infrequently (20% of trials), an arrow pointing upwards (stop signal) is presented after an arrow pointing left or right (go signal), indicating that the participant should withhold the response. The time window between the go signal and stop signal is individually adjusted based on performance, resulting in an error percentage of 50% for all participants. (duration 10 minutes)

**MRS scan.** We will collect a MRS scan, to able to directly assess the concentration of Glutamate in the striatum before and after the intervention (duration 8 minutes).

*Table 1. Healthy Diet: which Products are allowed and how often are they allowed?*

| Food group | Allowed | Per day 3 to 5 times allowed | Per week max. 3 times allowed | Not allowed |
| --- | --- | --- | --- | --- |
| Bread and breakfast cereals | Rye bread, rye crisp bread, whole-grain and brown bread, whole-grain muesli bread and brown bun.  Oatmeal.  Muesli and other whole-grain cereals with no added sugars.  Pancake made with whole-grain flour. |  | Croissant, currant bun. 1 slice sugar-loaf, currant-bread or raisin-bread.  1 bowl crunchy muesli, cornflakes, cruesli or other cereals with added sugar.  Pancake made with plain flour. | White bread, white currant-bread, white crisp bread. |
| Margarine/low-fat margarine on bread | Soft margarine, low-fat margarine, light bread spreads in a tub |  | Butter with at least 80% butterfat or margarine, for one slice of bread |  |
| Sandwich spreads or fillings | 100% peanut butter or nut butter with no added sugar.  Homemade hummus, fish, egg, (grilled) vegetables or fruit.  30+ cheese, soft goat cheese, cottage cheese, mozzarella and cream cheese. | Jam, honey, chocolate sprinkles/flakes or apple spread.  Chicken fillet, ham, luncheon meat, turkey fillet or 1 frankfurter.  Celery salad, cucumber salad, “sandwich spread” (spread with a salad dressing base), or pre-packaged hummus for one slice of bread | Chocolate spread or coconut bread.  Cured ham, salami, smoked beef, bacon.  40+, 45+, 50+ cheese for 1 slice. | Peanut and nut butter with added sugar.  Liver pâté and other  savory pre-packaged spreads (except celery and cucumber salad).  Feta. |
| Dairy | Buttermilk, skimmed milk, semi-skimmed milk, skimmed yoghurt, semi-skimmed yoghurt and skimmed soft curd cheese.  Alpro soya original products. |  | Full cream milk, full cream yoghurt, full cream soft curd cheese, custard, pudding and desserts, dairy and soya drinks with added sugars.  Plant-based protein drinks like soya drink (other than Alpro original), rice milk and almond milk. |  |
| Fruit | Fresh fruit, frozen fruit and drained fruit from a can or jar with no additives (like sugar). | 1 tablespoon fruit pulp (like apple sauce) or fruit from a can (e.g. 1 slice pineapple) | Bowl fruit from a can or fruit pulp. | Squeeze fruit snack |
| Meat (substitute) / fish / chicken / egg / legumes | (Sliced or minced) meat and poultry which is unprocessed and with no additives. Like lean minced meat, steak tartar, lean chuck roast, blade steak, chicken, turkey, steak and pork tenderloin.  Egg.  Fish (especially fatty fish, such as salmon or mackerel).  Natural tofu and tempeh.  Quorn pieces Des.  Legumes (dried and pure) from a can or jar without additives. |  | Processed meat and poultry like hamburgers, pork wrapped in bacon, marinated meat. Fatty meat, like pork belly, minced meat and lamb chop.  Croquette, minced-meat hot dog and other meat snacks.  Pre-packaged meat substitutes. | Legumes from a can or jar with added sugars. |
|  | *Note: your child can eat a maximum of 4 to 5 days per week meat, at least once a week fish and at least once a week a vegetarian alternative (e.g. egg, nuts, legumes and meat substitutes) with a warm meal.* | | | |
| Nuts | Nuts without coating (so without additives such as sugar) |  |  |  |
| Cooking fats | All sorts of oil, liquid bake, roast and deep fryer fat. |  | Hard margarine (sold in blocks), hard bake, roast and deep fryer fat and coconut oil. |  |
| Vegetables | All kinds of fresh vegetables (raw and cooked), frozen vegetables and vegetables from a can or jar without additives such as sugar. | 1 tablespoon corn from a can  3 olives  1 pickle | Portion vegetables from a can or glass with additives. | Vegetables in cream. |
| Potatoes / pasta / rice | Cooked or baked potatoes, homemade mashed potatoes, fries or potatoes baked in good fats.  Whole-grain pasta and couscous, brown rice.  Whole-grain tortilla. |  | Portion white rice, couscous or pasta. | Pre-packaged mashed potatoes or fries baked in hard deep fryer fat. |
| Sauces / seasoning / soup | Fresh and dried herb(mixes), onion, garlic, pepper and balsamic(vinegar).  Homemade soup | 1 tablespoon sauce (like ketchup, curry, mayonnaise with a maximum of 25% fat, garlic sauce, barbecue-sauce or honey)  1 tablespoon peanut sauce, soy sauce, kecap, mustard. | 1 tablespoon mayonnaise.  Instant soup. | Maggi (bottle).  Sambal.  Crème fraiche, cooking cream, sour cream or whipped cream.  Pre-packaged lettuce dressing.  Instant gravy.  Sugar. |
| Drinks | Water (bottled at source, tap, mineral) and tea without sugar (optional: with some semi-skimmed milk) | Glass of light soda or glass of lemonade without sugar, sweetener is allowed. | A glass of soda, lemonade, (thickened) juice or sport drink (note: 1 bottle contains 2 glasses) | Energy drink. |
| Snacks | Fresh and dried fruit.  Vegetables unlimited: e.g. carrot, slices cucumber, slices bell pepper or tomatoes.  1 tablespoon nuts.  Rye crisp bread or rice cracker (optional: with sandwich spread or filling from the column ‘allowed’)  Bread stick. | A few candies (e.g. licorice, wine gum, marshmallow).  Yoghurt raisins.  Piece of chocolate.  Small cookie (e.g. ginger cookie, biscuit, ‘’ladyfinger’’ or small slice of gingerbread)  Small ice lollipop. 1 scoop yoghurt or sorbet ice cream.  1 hand potato chips, popcorn or Japanese mix.  “Bitterbal” (small, spherical croquette), small Vietnamese spring roll (20 gram). | Mini candy bar.  Muesli bar.  Wrapped cookies. Portion (wrapped) ginger bread.  Almond round, pink glazed cake, slice of cake.  Lange ice lollipop, 1 scoop ice cream or slice ice cream cake.  Small bag or bowl potato chips. | All other cookies, candy, sweet and savory snacks. |
|  | *Your child can eat a snack 2 to 4 times per day. Your child can drink water and tea unlimited.* | | | |
